# Newborn's neural representation of instrumental and vocal music as revealed by fMRI: A dynamic effective brain connectivity study

**DOI:** 10.1002/hbm.26724

**Published:** 2024-07-12

**Authors:** Serafeim Loukas, Manuela Filippa, Joana Sa de Almeida, Andrew S. Boehringer, Cristina Borradori Tolsa, Francisca Barcos‐Munoz, Didier M. Grandjean, Dimitri van de Ville, Petra S. Hüppi

**Affiliations:** ^1^ Division of Development and Growth, Department of Pediatrics University of Geneva Geneva Switzerland; ^2^ Institute of Bioengineering, École Polytechnique Fédérale de Lausanne (EPFL) Lausanne Switzerland; ^3^ Swiss Center for Affective Sciences, Department of Psychology and Educational Sciences University of Geneva Geneva Switzerland; ^4^ Lemanic Neuroscience Doctoral School University of Geneva Geneva Switzerland; ^5^ Division of Pediatric Intensive Care and Neonatology, Department of Women, Children and Adolescents University Hospital of Geneva Geneva Switzerland; ^6^ Department of Radiology and Medical Informatics University of Geneva Geneva Switzerland

**Keywords:** instrumental music, music perception, newborns, prematurity, vocal music

## Abstract

Music is ubiquitous, both in its instrumental and vocal forms. While speech perception at birth has been at the core of an extensive corpus of research, the origins of the ability to discriminate instrumental or vocal melodies is still not well investigated. In previous studies comparing vocal and musical perception, the vocal stimuli were mainly related to speaking, including language, and not to the non‐language singing voice. In the present study, to better compare a melodic instrumental line with the voice, we used singing as a comparison stimulus, to reduce the dissimilarities between the two stimuli as much as possible, separating language perception from vocal musical perception. In the present study, 45 newborns were scanned, 10 full‐term born infants and 35 preterm infants at term‐equivalent age (mean gestational age at test = 40.17 weeks, SD = 0.44) using functional magnetic resonance imaging while listening to five melodies played by a musical instrument (flute) or sung by a female voice. To examine the dynamic task‐based effective connectivity, we employed a psychophysiological interaction of co‐activation patterns (PPI‐CAPs) analysis, using the auditory cortices as seed region, to investigate moment‐to‐moment changes in task‐driven modulation of cortical activity during an fMRI task. Our findings reveal condition‐specific, dynamically occurring patterns of co‐activation (PPI‐CAPs). During the vocal condition, the auditory cortex co‐activates with the sensorimotor and salience networks, while during the instrumental condition, it co‐activates with the visual cortex and the superior frontal cortex. Our results show that the vocal stimulus elicits sensorimotor aspects of the auditory perception and is processed as a more salient stimulus while the instrumental condition activated higher‐order cognitive and visuo‐spatial networks. Common neural signatures for both auditory stimuli were found in the precuneus and posterior cingulate gyrus. Finally, this study adds knowledge on the dynamic brain connectivity underlying the newborns capability of early and specialized auditory processing, highlighting the relevance of dynamic approaches to study brain function in newborn populations.

## Graphical abstract

Newborns listened to a vocal or instrumental melody. During the vocal condition, sensorimotor and salience network were activated, while during instrumental condition, visual and superior frontal cortex. Common neural signatures for vocal and instrumental melodies were also found, indicating a perceived musicality of the sound stimuli.

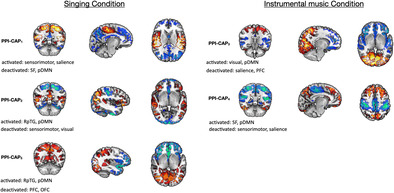

## 1 INTRODUCTION

The number of similarities between music and speech moved several authors to both highlight the parallels and disentangle the specificities of the two, especially in their neural perceptive correlates (Patel, 2010; Peretz et al., 2015). In adult studies, a meta‐analysis (Schirmer et al., 2012), confirming the existing proposal of temporal lobe specialization, specifically asserted that vocalizations, more likely than music, can activate the left primary/secondary auditory cortex, left superior temporal sulcus, and medial temporal gyrus, while music showed no more brain activations than voices. However, as authors claimed, the importance of variability of the included stimuli needs to be considered for further analyses (for music: solo instruments, orchestras, multiple timbers, diverse music genres; for voice: linguistic/nonlinguistic vocalizations, syllables, tones; for controls: sound environmental stimuli, natural/artificial sounds and noise). To further compare singing and music, previous studies proposed to minimize the difference between the two stimuli (voices and instruments) by using singing voices as a comparison to music (Schön et al., 2010; Whitehead & Armony, 2018). In the present investigation on the origins of the neural perception of voice and instruments, we aimed to further simplify the experimental paradigm, and we proposed to newborns the same five melodies either sung by a female voice or played by a musical instrument.

By using singing and instrumental stimuli with similar basic acoustical parameters (melodic line, pitch, intensity), the spectral quality of the sound, given by its overtones and perceived as a timbre, could be one of the main acoustic cues allowing to distinguish the two stimuli. In particular, by using an instrument very similar to the human voice, such as an Indian flute, we aim to investigate the newborn's ability to distinguish between the two stimuli would be based on very specific abilities in timbre discrimination, which were shown to be already present at birth (Háden et al., 2009).

Newborn infants, far from being tabula rasa, possess important auditory skills that they developed in utero (Partanen et al., 2013) that enable them, after birth, to orient in a world of multiple environmental auditory stimulations. These skills form the basis for the development of their language, social and communicative skills (Huotilainen, 2010).

### 1.1 Newborns' music processing

Right after birth newborns have been shown to already possess early music processing capacities. They can represent pitch trends within sound sequences, process them in a predictive manner (Haden et al., 2015; Lee et al., 2009), and can discriminate between different rhythmical patterns (Ramus, 2002). They are also sensitive to tempo changes in the presentation of a series of notes (Haden et al., 2015; Lee et al., 2009) and can detect consonance versus dissonance and minor/major chord changes (Perani et al., 2010; Virtala et al., 2013). Also, importantly for the present study, they can perceive small differences in musical timbres (Háden et al., 2009). Whether there is a lateralization regarding music listening processing in infants is still an open question, given the existence of conflicting results (Kotilahti et al., 2010). Evidence has shown that newborns can also recognize complex streams of sounds, such as familiar vocal melodies, musical excerpts, and nursery rhymes, to which they were exposed during the last trimester of pregnancy (Granier‐Deferre et al., 2011; Hepper & Shahidullah, 1994; Partanen et al., 2013). This memory formation has been traced in preterm newborns as well: when preterm infants are exposed daily to a specific instrumental music, for a minimum of 2 weeks during Neonatal Intensive Care Unit stay (NICU), they recognize it as familiar and pleasant when relistening weeks later at their expected date of birth (Lordier, Loukas, et al., 2019). These musical memories, formed with the repeated exposure to the musical stimulus, are functionally consolidated, as shown by post stimulus resting state functional connectivity and imply memory retrieval and associative memory already during the newborn period, as previously suggested by physiological responses to repeated musical stimuli to which fetuses were exposed in the prenatal period (Loukas et al., 2022). Studies evaluating the beneficial effect of auditory enrichment through vocal and instrumental music in early care for preterm newborns are in progress and the design of such stimuli rely on a better understanding of brain processing capacities in the newborn.

### 1.2 Newborns are oriented to voices

Within the multiple auditory stimulations of their environment, newborns specifically orient to human voices as salient stimuli, and they possess a strong endowment to process speech (Dehaene‐Lambertz et al., 2010): they can process phonetic differences between syllables, discriminate between different speakers, that is, mother versus stranger (Adam‐Darque et al., 2020; Perani et al., 2010), and they are sensitive to small changes in prosody (Sambeth et al., 2008).

Even though neural substrates for spoken language have been found in both hemispheres, including the left and right temporal cortices and the left inferior frontal cortex (Perani et al., 2011), newborns appear to have a left‐hemisphere predominance in processing speech (Pena et al., 2003). Specifically, they process regularities in speech in left inferior frontal areas, including Broca's area, the same areas dedicated to language processing in adults (Gervain & Mehler, 2010).

However, it is important to note that emotional characteristics of speech (Cheng et al., 2012) and novelty‐related responses (Beauchemin et al., 2011; Perani et al., 2011) are primarily processed in the right hemisphere. Interestingly, newborns can distinguish between singing and speaking, even when they have the same linguistic content, and are more engaged by rich prosodies (Sambeth et al., 2008).

Similarly, there is convincing evidence that, as with adults (Schirmer et al., 2012), infants possess distinct abilities to process and distinguish spoken voice from music (Best et al., 1982).

Dehaene‐Lambertz et al. (2010) showed that speech inputs are preferentially treated in the left side. More specifically, at the level of the planum temporale there is a left‐hemispheric advantage for speech relative to music, which results in bilateral patterns of activation in the same area. Similar results have been found in adults (Callan et al., 2006).

However, the aforementioned studies did not reveal the specificities between instrumental versus vocal music processing in newborns. No differences were found in a single preliminary study using EEG and ECG for instrumental versus vocal music perception in newborns, born to depressed or non‐depressed mothers (Hernandez‐Reif et al., 2006), but the authors claimed the need for further studies.

To address this question, we specifically explored the functional differences regarding vocal and instrumental music processing in the newborn infant. Singing presents acoustic qualities which are different from speech in terms of rhythmical segmentation, spectral properties, variability in tempo, pitch, and intensity (Livingstone et al., 2013). However, singing shares the same medium with the speech, the voice, and in conveying emotions they show several common acoustic features (Livingstone et al., 2013). On the other hand, singing has many elements in common with music. The singing voice is embedded in a precise rhythmical structure, which can allow the formation of expectations in newborns (Haden et al., 2015; Lee et al., 2009) and through repetition it can enable and facilitate the memorization of sound sequences (Falk et al., 2021).

It would therefore be of interest to evaluate dynamic functional differences regarding vocal and instrumental music processing in the newborn infant. This scientific question is the main aim of the present study.

## 2 MATERIALS AND METHODS

### 2.1 Population

A cohort of 54 very preterm (gestational age [GA] at birth <32 weeks) and 24 full‐term infants, were recruited at the neonatal unit of the University Hospitals of Geneva (HUG), Switzerland, from 2017 to 2021. The inclusion criteria for preterm newborns were very strict, to assure a homogeneity within the population and exclusion criteria for all newborns included major brain lesions detected on the MRI, such as high‐grade intraventricular hemorrhage (grade III or IV), as well as micro‐ or macrocephaly, hydrocephaly, leukomalacia and congenital syndromes. Six initially eligible and recruited preterm infants were finally not included in the study due to a posteriori parental withdraw (5 very preterm infants) or diagnosis of genetic syndromes (1 very preterm infant). Also excluded from the analysis were infants whose MRI protocol acquisition was incomplete or that had major motion artefacts.

The final sample comprised 45 newborns, that were scanned at term equivalent age, at a mean of 40.17 (SD = 0.44) weeks GA. From these, 35 were born very preterm and were scanned at term‐equivalent age (*m* = 40.17 weeks GA SD = 0.55) and 10 were born at full‐term age (*m* = 40.18, SD = 0.75), and were scanned few days after birth.

The postnatal age at the time of testing ranged from a mean of 77 days for preterm infants (11 weeks, SD = 1.6 weeks), to a mean of 2.7 days for term newborns. As reported in Table 1, when comparing the GA at birth with the GA at test, preterm infants' postnatal age at the time of testing is not homogeneous between the included newborns. However, no significant differences were found between full‐term infants and very preterm infants regarding gestational age at the MRI (*p* = 0.945). The population characteristics are presented in Table 1.

**TABLE 1:** Clinical characteristics of the included newborns.

Clinical characteristics	Preterm (PT)	Full‐term (FT)	All infants	*p* value*
	*n* = 35	*n* = 10	*n* = 45	PT vs. FT
Gestational age at birth, weeks, mean (SD)	29.19 (±1.9)	39.79 (±2.3)	31.54	0.001*
Gestational age at birth, weeks, range	24^1/7^ to 31^6/7^	38^6/7^ to 40^6/7^	24^1/7^ to 40^6/7^	
Gestational age at MRI scan, weeks, mean (SD)	40.17 (±0.55)	40.18 (±0.75)	40.17 (±0.44)	0.945
Gestational age at MRI scan, weeks, range	38^5/7^ to 41^1/7^	39^0/7^ to 41^3/7^	38^5/7^ to 41^3/7^	
Sex: female (%)/male (%)	19 (42)/16 (36)	6 (13)/4 (9)	25 (56)/20 (44)	0.748
Socio‐economic score, mean (SD)	4.17 (±2.7)	4 (±3.0)	4.13 (±2.7)	
Birth weight, gram, mean (SD)	1214.7 (±391.2)	3326.5 (±406.1)	1684 (±969.7)	0.001*
Birth height, centimeter, mean (SD)	37.9 (±4.2)	50.6 (±1.8)	40.8 (±6.5)	0.001*
Birth head circumference (cm), mean (SD)	26.7 (±2.7)	34.9 (±1.17)	28.5 (±4.2)	0.001*
APGAR score 1 min, mean (SD)	5.17 (±3.14)	8.6 (±2.0)	5.93 (±3.2)	0.002*
APGAR score 5 min mean (SD)	7.97 (±1.9)	9.7 (±0.68)	8.36 (±1.9)	0.008*
Intrauterine Growth Restriction, *n* (%)	6 (13)	0	6 (13)	0.16
Neonatal asphyxia, *n* (%)	0	0	0	1
Bronchopulmonary dysplasia, *n* (%)	14 (31)	0	14 (31)	0.016*
Sepsis, *n* (%)	8 (18)	0	8 (18)	0.095*
Intraventricular hemorrhage (grade 1), *n* (%)	6 (13)	0	6 (13)	0.16*

*Note*: *Group‐characteristics were compared using independent samples T‐test for continuous variables and chi‐squared test for categorical variables.

The study was approved by the local Research Ethical Committee and written parental consent was obtained before the infants' participation in the study. All subjects underwent an fMRI examination at term‐equivalent‐age (mean GA [weeks] at scan PT: 40.17 and FT: 40.18). Infants whose MRI protocol acquisition was incomplete, not comprising a T2‐weighted image and/or task‐based functional magnetic resonance (fMRI) sequence, or that presented major focal brain lesions were excluded from the analysis.

### 2.2 fMRI acquisition

Newborns underwent a 3 T Siemens MRI scan at term‐equivalent‐age (TEA), comprising a task‐based fMRI and T2‐weighted structural image sequence. T2‐weighted structural images were acquired using the following parameters: 113 coronal slices, TR = 4990 ms, TE = 160 ms, flip angle = 150°, voxel size = 0.8 × 0.8 × 1.2 mm^3^. fMRI data were obtained by means of T2*‐weighted gradient‐echo EPI images with the following parameters 590 images, TR = 700 ms, TE = 30 ms, 36 slices, voxel size = 2.5 × 2.5 × 2.5 mm^3^, flip angle = 60°, multi‐band factor = 4.

All newborns were scanned after receiving breast or formula feeding, during natural sleep, and no sedation was used. To prevent motion artefacts during data acquisition, newborns were placed in a vacuum mattress before entering the fMRI machine. MR‐compatible headphones were used (MR confon, Magdeburg, Germany) to deliver the different auditory stimuli and protect infants from the scanner's noise. The fMRI acquisition consisted of several sequences, totaling a maximum of 45 min. During data acquisition, heart rate and oxygen saturation were monitored in infants.

### 2.3 Stimuli

During the task‐based fMRI experiment, newborns were listening to a melody (without words) sung either by a female voice, or played by a musical instrument (Indian flute).

The entire melody was divided in five extracts lasting 8 s each (two examples of sonogram for the instrumental stimulus and two for the vocal stimulus are provided in Figures S12–S15). The mean pitch of the instrumental extracts is 319.2 Hz (range 320–360 Hz) and of the vocal extracts is 322 Hz (range, 319–365). No significant differences were found between the instrumental and vocal extracts in terms of intensity, mean pitch, and range.

A silence condition and a noise condition (white noise) completed the experimental sequence. The order of the conditions was pseudo‐randomized and each condition was repeated 5 times in a block design setup. To control for differences in loudness, all the stimuli were normalized using the MP3Gain Express 2.4.0 software.

### 2.4 MRI data processing

fMRI data were preprocessed using SPM12 (Wellcome Department of Imaging Neuroscience) in MATLAB R2022a. For each participant, the MRI scans were first spatially realigned and then co‐registered to the structural images in the subject space. Next, the fMRI volumes were normalized using an appropriate study‐specific neonatal template and finally, smoothed with a Gaussian filter of full width at half maximum (FWHM) of 6 mm.

### 2.5 Head motion & quality control

All fMRI volumes with a frame‐wise displacement (Power et al., 2014) >0.5 mm or with a rate of BOLD signal changes across the entire brain (spatial standard deviation of successive difference frames, DVARS) >3% were removed, along with the two previous and two successive images. Moreover, as a second robust quality control step, we retained good frames that constitute blocks of consecutive good volumes that are long enough to include at least one repetition of each fMRI experimental condition. This ensures that the retained frames are balanced with respect to the experimental conditions' appearances/occurrences. The remaining retained images were included for further analysis.

### 2.6 Data analysis

To explore the dynamic, moment‐to‐moment, task‐based changes in brain functional connectivity during the fMRI task involving both instrumental (Indian flute) and female voice conditions, we employed a psychophysiological interaction of co‐activation patterns (PPI‐CAPs) analysis (Freitas et al., 2020) on the subject‐concatenated data to obtain group‐level CAPs. This innovative dynamic method allows us to explore whether moment‐to‐moment brain functional‐connectivity differs when listening to an instrumental melody versus a singing melody in infants at TEA (by analyzing the temporal characteristics, e.g., PPI effect of the CAPs).

Briefly, the PPI‐CAPs method selects moments in which a seed is highly active and then clusters (CAPs analysis as first step) these frames based on their activation patterns, allowing positive and negative polarities of the same pattern to be clustered together (i.e., brain activity patterns with completely opposite signs, for more details see Sambeth et al., 2008). The CAPs are the representative dynamic brain activity patterns that were mainly occurring across the experimental run (MRI scanning). Next, each obtained PPI‐CAP was tested whether it varies according to the seed activity, the task, or an interaction between the two (i.e., the PPI effect). In this study, we selected the combined left and right auditory cortices as a seed region.

To identify the optimal number of PPI‐CAPs (number of clustering centroids), we performed a consensus clustering analysis (Monti et al., 2003). This approach applies K‐means clustering on several subsamples of the data and calculates the consensus matrix for different numbers of clusters each time. In the present study, we explored the *k* range from 3 to 8 to identify the optimal number of clusters for our data using 10 random subsamples folds including 80% of the subjects for every *k*. The optimal number of clusters can then be inferred by the ordered matrix, as well as the proportion of ambiguous clustering (PAC) metric of the consensus matrix. Based on the consensus clustering, the optimal number of PPI‐CAPs was determined to be *k* = 5 (see Figures S1 and S2). Once the final *k* was identified, the final clustering step was performed using 100 replicates to ensure clustering robustness.

### 2.7 Network assignment

After the five PPI‐CAPs were obtained in the final clustering step (*k* = 5), we identified the regions and networks highlighted in each pattern by overlaying ICA‐derived functional networks identified in a previous study (Lordier, Meskaldji, et al., 2019): Visual, Sensorimotor, Superior frontal, posterior DMN (Posterior Cingulate Cortex & Precuneus), Right posterior temporal cortex (RpTG), Prefrontal Cortex (PFC), Left posterior temporal cortex (LpTG), Salience, Orbitofrontal (OFC) (see Figure S3 and Table S1). In this study, we further merged the Posterior Cingulate Cortex and Precuneus components to form the posterior DMN (pDMN) component.

In more detail, the network identification was achieved by calculating the proportion of each functional network that was activated or deactivated in each PPI‐CAP. The two most activated or deactivated ICA networks defined the activity “profile” of each of our five PPI‐CAP maps (see Figure S4 and Table S1).

### 2.8 Effect assessment

For each PPI‐CAP, we tested the task effect (instrumental versus vocal stimulus) in presence of the seed (auditory) activation, and one seed‐task interaction effect (PPI effect). If a PPI‐CAP is characterized by a strong main or interaction effect, the polarity of its frames tends to correlate with the sign of that effect for the same time points (i.e., the sign of a PPI‐CAP switches often in the same way as each of the underlying effect) (Freitas et al., 2020). Confusion matrices can be constructed to visualize this information for each effect and PPI‐CAP. Strong correlations are represented by higher values on one of the confusion matrix diagonals (main or anti‐diagonal). The strength of this relationship can be measured by the confusion matrix's determinant (Freitas et al., 2020). For details see Figure 2.

### 2.9 Significance assessment

To test whether the confusion matrix's determinant value was significant, random permutations were employed. The effect of interest's (main or interaction) labels were permuted and the determinant was re‐estimated at each iteration to define the null distribution for the given effect. *p* values were calculated by estimating where the observed real determinant value lies within the null distribution. In this study, we performed 2000 random permutations for each test, and we report significant results at Bonferroni level of α = 0.01 (0.05/5), correcting for the number of PPI‐CAPs.

## 3 RESULTS

### 3.1 Dynamic brain patterns derived from auditory‐based PPI‐CAPs analysis

**FIGURE 1:**
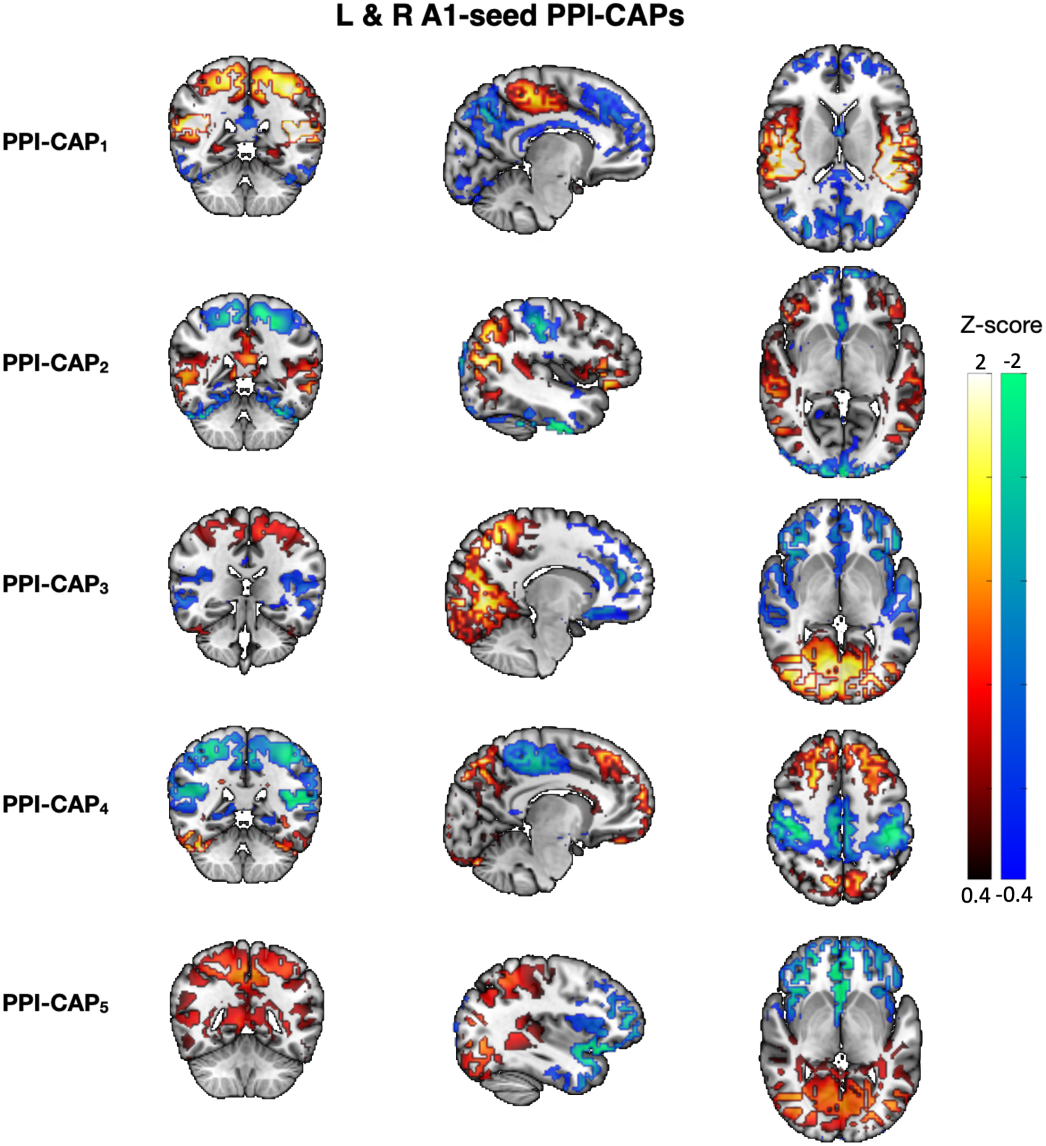
CAPs using the auditory cortex as seed. Each row corresponds to a CAP and the color bar shows z‐score values. Views focused at [5 1 25] MNI coordinates. PPI‐CAP effects are evaluated as a second step.

To unveil the neural networks involved in vocal and instrumental music processing, we employed the dynamic PPI‐CAPs analysis that yielded the five most prominent dynamic patterns of brain activity (see Figure 1). PPI‐CAP 1 corresponds to an activated sensorimotor and salience network, and deactivated superior frontal (SF, bilateral medial superior frontal) and posterior DMN (pDMN: precuneus, posterior cingulate gyrus). PPI‐CAP 2 includes activated right posterior temporal gyrus (RpTG) and pDMN, and deactivated sensorimotor and visual networks. PPI‐CAP 3 consists of activated visual and pDMN networks and deactivated salience and superior frontal (PFC, superior, middle and inferior frontal gyri). PPI‐CAP 4 is assigned to an activated SF and pDMN and deactivated sensorimotor and salience networks. Finally, PPI‐CAP 5 is characterized by an activated RpTG and pDMN, and deactivated PFC and orbitofrontal (OFC) networks (see Figures 1 and S4; upper panel). Some of the PPI‐CAPs appear to share similarities however, by definition, they are unique dynamic brain activity patterns (unique clustering centroids). Here, we interpret our results based on the aforementioned network assignment.

### 3.2 Co‐activations patterns

**FIGURE 2:**
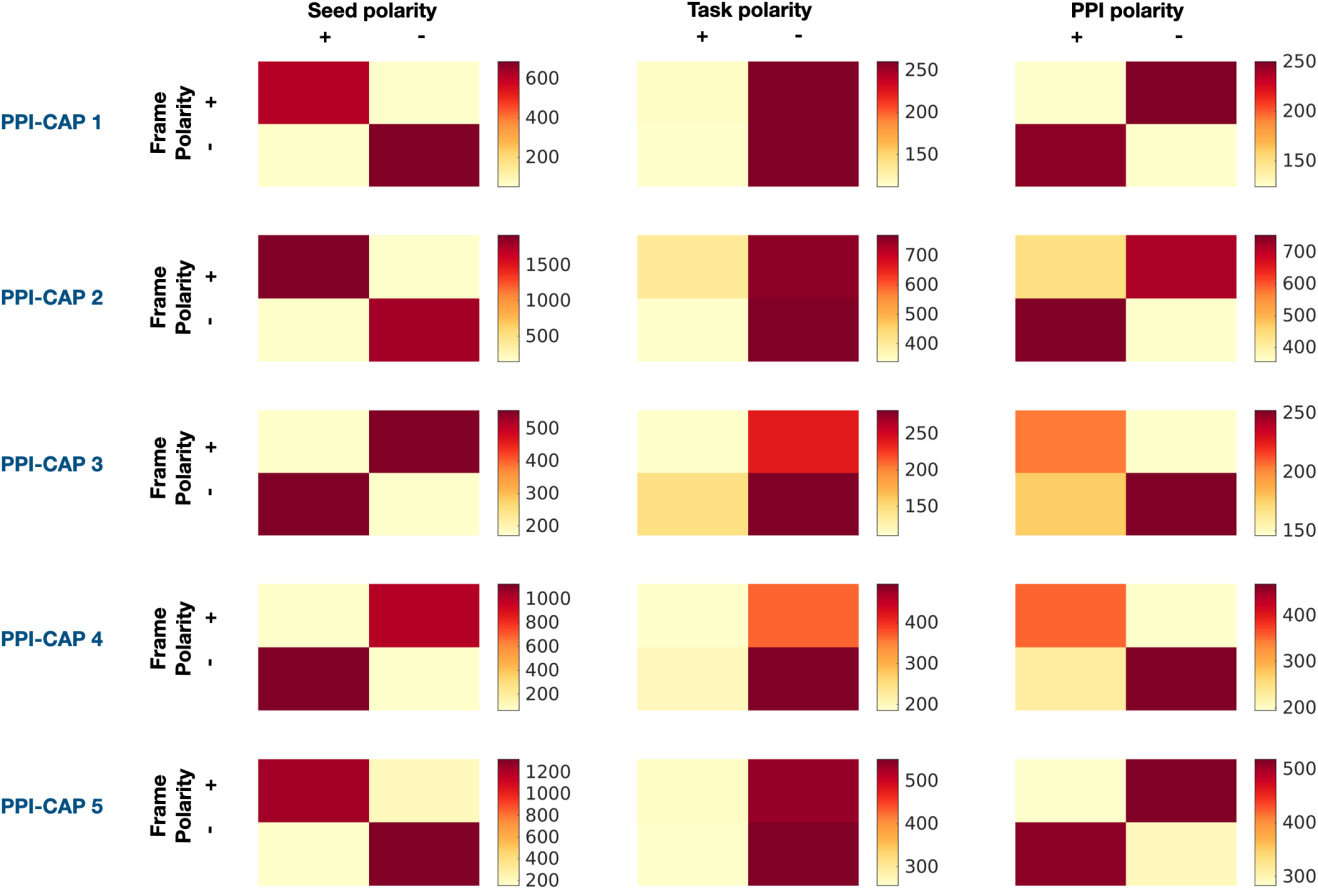
PPI‐CAPs analysis reveals main and interaction effects. Each row corresponds to a PPI‐CAP and each column to a specific effect (seed, task or PPI effect, respectively). Main and interaction effects can be revealed using confusion matrices that depict how often the sign of a PPI‐CAP switches in the same way as each of the underlying effects. The signs for each effect were defined as follows: Seed—positive and negative signs correspond to frames when the seed (auditory cortex) was activated or deactivated, respectively; Task—positive signs correspond to the Instrumental music condition while negative signs correspond to moments of Singing Condition; PPI—Interaction signs are calculated as element‐by‐element multiplication of the main effect signs. Light yellow indicates the lowest number of frames, while dark red indicates the highest number of frames. Null distribution and the exact *p* values of these effects are shown in Figure S3.

To define the specific neural processing of vocal and instrumental music listening, for each PPI‐CAP we tested two main effects, seed (auditory) and task (vocal, instrumental), and an interaction effect. This was done by exploring whether the flipping of each pattern correlates with the sign of each effect, as described in the Section 2. The resulting confusion matrices are presented in Figure 2. Each row corresponds to a PPI‐CAP and each column to a specific effect. Permutation testing was used to assess which effects were significant. The *p* values and permutation null distributions are presented in Figure S5.

### 3.3 Dynamic co‐activations patterns with the seed (auditory cortex) and the task (instrumental vs. vocal)

Based on the permutation testing, all PPI‐CAPs showed a significant seed effect (*p* < 0.001, see Figure S5). The proportion of activations or deactivations of the identified networks is presented in Figure S5. The diagonal pattern in the confusion matrices for PPI‐CAP 1, 2 and 5 (see first column in Figure 2), suggest that these patterns are positively correlated with the seed (active auditory cortex). On the other hand, PPI‐CAP 3 and 4 are negatively correlated with the seed (deactivated auditory cortex). No significant task effect, vocal versus instrumental music, was found following Bonferroni correction.

### 3.4 Dynamic co‐activation patterns based on seed (auditory) and task (instrumental/vocal) interaction

**FIGURE 3:**
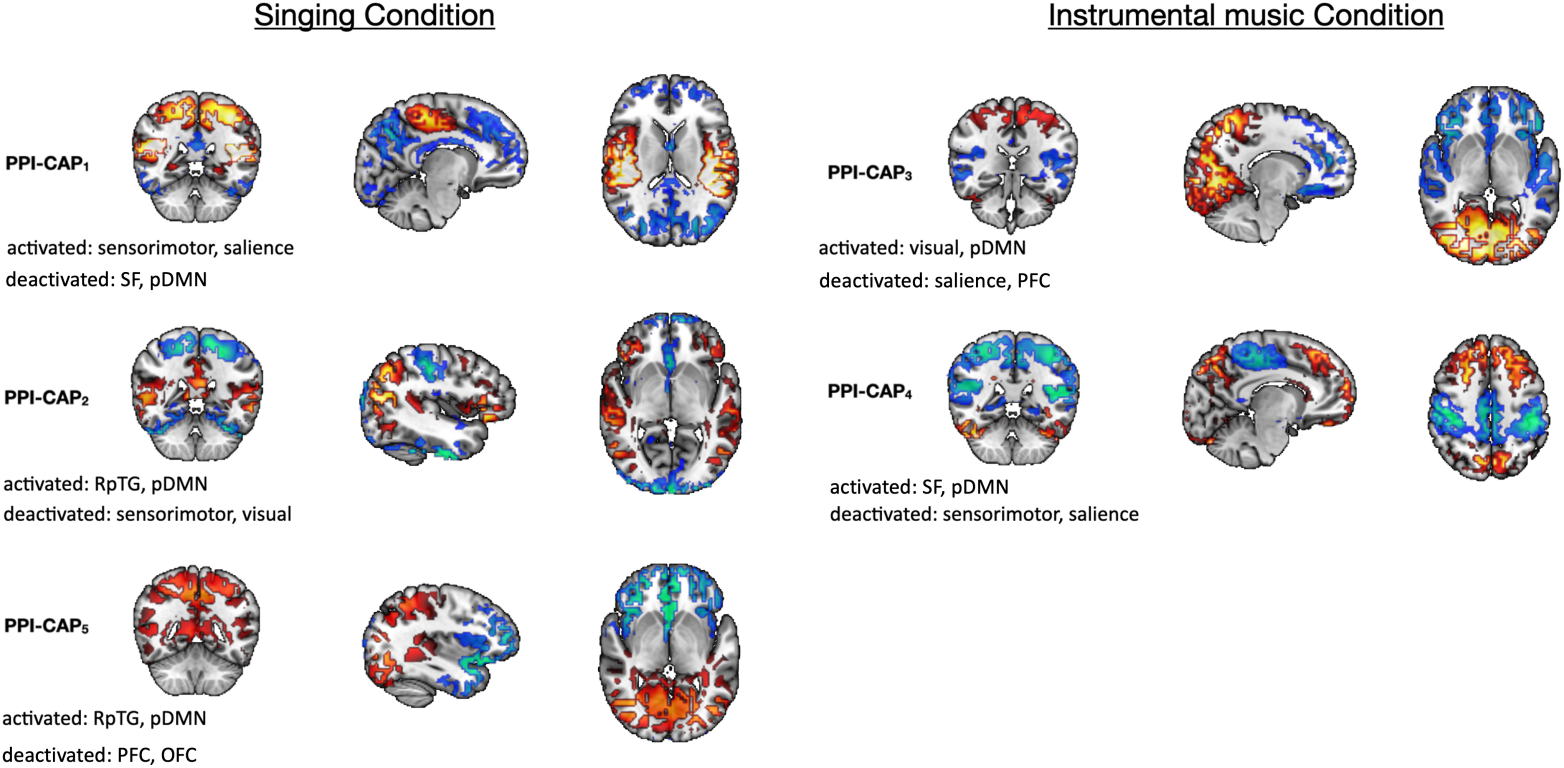
Summary of the PPI effects: dynamic seed‐based connectivity for singing versus instrumental music condition. OFC (medial orbital cortex and gyrus rectus, inferior temporopolar region, planum polare), pDMN (posterior cingulate cortex: bilateral precuneus gyrus, posterior cingulate cortex, and bilateral precuneus), RpTG (right posterior superior and middle temporal gyrus), Salience (Insula, perigenual cingulate cortex), Sensorimotor (bilateral pre and post central gyrus), SF (bilateral medial and superior frontal cortex), PFC (prefrontal cortex), Visual (bilateral occipital lobe).

PPI‐CAP 1, 2, and 5 show a significant negative interaction between the seed and task (PPI effect) (all, *p* < 0.001, see third column in Figure S5). This suggests that these dynamic patterns of brain activity (Figure 1) correlate with the seed more during the Singing condition compared to the Instrumental music condition. In contrast, PPI‐CAP 3 and 4 have a positive interaction between seed and task (PPI effect, *p* < 0.001, Figure S5), meaning that these patterns are more correlated with the seed during the Instrumental music condition, compared to the Singing condition (i.e., are more often expressed in their current polarity during the Instrumental music condition and in the presence of seed activity, see Figure 3 for Results summary). The pDMN network is present among all the PPI‐CAPs, except for PPI‐CAP 1, that deactivates in the presence of the vocal stimulus (for details see Figure 3 and Section 4) and its activation is common to both instrumental and vocal stimuli.

As an additional analysis, the contrast between the mother's and stranger's singing voices was performed to differentiate specific activations elicited by the two auditory stimuli. No significant differences were found, except for a specific activation of PPI‐CAP 2 for the mother's voice and PPI‐CAP 5 for the stranger's voice. However, since the activated areas in the two CAPs were similar (right posterior temporal gyrus and pDMN) and as it was not the main aim of the present analysis, we conducted the general analyses with both stimuli combined into a single variable called ‘voice’. The supplementary analyses are presented in Figures S5 and S6.

## 4 DISCUSSION

The present findings show specific dynamic co‐activation patterns between the auditory cortex and higher order cortical networks—here named as PPI‐CAPs analysis—when newborns are exposed to different auditory tasks, more specifically when they listen to either vocal or instrumental music. First, common neural signatures of dynamic brain activity for both stimuli, voice and instrument, are evidenced, namely the pDMN network, here comprised of precuneus and posterior cingulate, which is involved among all the PPI‐CAPs, mostly co‐activating during both tasks and deactivating only when the somatomotor area and the salience network activate during the vocal melody presentation. In the literature, at a functional level, precuneus and posterior cingulate gyrus have been shown to be activated during basic melodic and harmonic information in the auditory stream (Spada et al., 2014), as well as when words or musical stimuli were eliciting emotions (Koelsch, 2018). These areas, in particular posterior cingulate, are also involved in pitch and familiarity perception (Castro et al., 2020; Platel et al., 1997). The activation of these regions could thus represent a common neural signature for melodic auditory stimuli, independently of being vocal or instrumental. Furthermore, a recent study of our group has shown that preterm infants that benefit from an early music intervention during neonatal intensive care stay (NICU) presented a longitudinally increased cortical maturation of the precuneus and posterior cingulate gyrus, in comparison with the control group (de Almeida et al., 2023), further supporting that these regions are elicited during music processing already early in life.

When compared to the instrumental music condition, the present results indicate that the vocal condition elicited specific brain activations. Namely, the salience and sensorimotor networks are more highly co‐activated in the singing condition than in the instrumental one. These results suggest that the vocal melody may be perceived as a more salient stimulus to newborns compared to a similar instrumental melody. This could be influenced by the fact that premature infants, part of the cohort studied, were more exposed to air transmitted vocal stimuli than full term infants. Whether this preference is due to the familiarity of the stimulus, with the singing voice being a more prevalent element in the everyday prenatal environment than the flute, remains unknown. The aforementioned finding is consistent with previous research, as it is known that speech perception sustains an early activation of motor representations (Lévêque & Schön, 2013). Imitation is one of the key mechanisms already present in the newborn period, and crucial to infants' vocal and motor development (Meltzoff et al., 2018; Simpson et al., 2014). Importantly, fMRI studies in preterm newborns listening to motherese vocal stimuli have shown similar motor activations (Adam‐Darque et al., 2020). Not only listening to speech, but also to non‐speech vocal sounds, such as cough or laughter, has been shown to activate the same motor regions as speech (Chang et al., 2009), indicating an early brain specialization also for emotional vocal stimuli. Moreover, emotional vocalizations activated the premotor cortex, bilaterally (Warren et al., 2006), which has also been observed during perception of sung syllables (Schön et al., 2010). The current dynamic functional connectivity analysis is in line with previously reported data focusing on EEG beta and mu brain oscillations, where authors reported that perceiving a singing voice induced a stronger sensorimotor activity than a non‐vocal melody (Lévêque & Schön, 2013). In addition, a resting‐state fMRI pilot study evaluating the effect of music therapy (vocal singing) on preterm infants, in comparison to standard care, showed to induce a stronger functional connectivity in supplementary motor regions (Haslbeck et al., 2020). Interestingly, in PPI‐CAP1, where sensorimotor and salience networks are more highly co‐activated when compared to instrumental stimuli, there was a concomitant deactivation of the superior frontal and pDMN networks. One interpretation is that when there is a sensorimotor and a selectively attentive response (salience network) activation, there is a concomitant deactivation of the DMN, which is typically activated during a resting condition, in which people do not engage intentionally in task activities. Indeed, the salience network has been shown to dynamically control changes of activity in other networks and is typically coupled with deactivation of the DMN, in order to enable efficient task performance (Bonnelle et al., 2012).

The perception of the vocal melody also activates the RpTG in two PPI‐CAPs. This is not the case during the instrumental melody processing. This area has been shown to be implicated not only in language processing but mostly in social perception. It has been shown to play a critical role in integrating different types of incoming information in order to give it a meaning, and it has been suggested to be dysfunctional in infants with autism (Boddaert & Zilbovicius, 2002; Jou et al., 2010). The stronger co‐activation of this area observed during the vocal melody in comparison to the instrumental stimulus can suggest that important functions such as language and social perception might be mediated by the vocal stimulus even when lacking speech content. Moreover, this specific lateralized activation in newborns, which in adults is essential for pitch perception (Warrier & Zatorre, 2004), could indicate that at this age the sensitivity for the pitch perception may be mediated by the vocal presentation.

Finally, in comparison to the vocal melody, the instrumental melody predominantly activates visual (PPI‐CAP3) and superior and medial frontal cortex (PPI‐CAP4), with a concomitant deactivation of the prefrontal cortex (PPI‐CAP3), sensorimotor areas (PPI‐CAP4) and the salience network (PPI‐CAP 3–4), which, inversely, were activated during the vocal melody. The visual and the superior and medial frontal cortex are regions both known to be activated during music listening (Janata et al., 2002; Särkämö et al., 2013). The superior and medial frontal cortex are implicated in higher cognitive functions and working memory, namely regarding spatial cognition (Boisgueheneuc et al., 2006) and have been shown to be activated during familiar music listening (Freitas et al., 2018). This suggests that instrumental music listening activates a visual–spatial processing dimension more strongly in comparison to voice. Furthermore, visual imagery is a crucial element in the music listening experience and might be one of the key mechanisms by which music induces emotions in listeners (Juslin & Västfjäll, 2008; Küssner & Eerola, 2019). Research on cross‐modal music perception demonstrates a clear correspondence between auditory and visual perception for musical stimuli (Küssner & Leech‐Wilkinson, 2014) as such defining a multisensory experience. Visual imagery in response to musical stimulus is not only the potential mechanism linking music and emotions, which could explain our recent findings on the impact of early music exposure to the maturation of newborn's emotional processing areas (de Almeida et al., 2020), but it also supports mental processes (Alley & Greene, 2008).

In the present study, these visual areas were largely concurrently co‐activated with pDMN in response to instrumental, but not to vocal music. It is known that the pDMN, here including the precuneus and the posterior cingulate gyrus, supports the activation of the so‐called mind‐wandering phenomenon (Christoff et al., 2009). In adults, visual imagery and mind‐wandering in music are strictly linked (Taruffi et al., 2017) and constitute key mechanisms for eliciting musical emotions. The present results demonstrated, for the first time, that this co‐activation pattern is highly active in the neonatal period in response to the instrumental but not vocal music. Interestingly, the pDMN, including posterior cingulate cortex and precuneus among others, are brain areas affected by prematurity and recent data indicate both a decreased functional connectivity in preterm infants compared to term born newborns and the potential of music to improve functional connectivity to the precuneus (Lordier, Meskaldji, et al., 2019) and multisensory processing in general. As the music intervention in this prior study was instrumental only the current data on vocal and instrumental specific brain co‐activation patterns will allow to design such music interventions in more brain‐based approach.

## 5 CONCLUSIONS

Our study is the first fMRI research aiming to evaluate dynamic functional differences regarding vocal and instrumental music processing in the newborn infant.

Vocal and instrumental melodies activate common neural signatures in newborns, comprising namely regions implicated in pitch and emotional processing. The dynamic aspects of the melody seem to activate regions responsible for a sensorimotor experience when presented by the vocal melody, while it activates visual imagery and a mind‐wandering experience when played by a musical instrument. Interestingly, the vocal melody seems to be perceived as a more salient stimulus to the newborn in comparison to a similar instrumental melody.

This study reveals that newborns process vocal and instrumental melodies differently, suggesting that each stimulus elicits specialized brain processing already early in life. The present results can pave the way for early and personalized auditory interventions in the neonatal period, based on the different brain activations induced from the two types of stimuli.

## 6 LIMITATIONS

One limitation of the study is the modest sample size. The specificity of the neonatal population imposes a difficult recruitment process, as well as diverse technical limitations such as motion during the MRI acquisition. For this reason, we have combined in our analysis both the full‐term and preterm newborns at term‐equivalent age, independently of the age at birth.

Thus, one of the main limitations of the current study is the heterogeneity of the included population, comprising both preterm (*n* = 35) and term (*n* = 10) newborns. Due to our limited sample size and to the multiple difficulties in recruiting such population, we decided to merge the two populations to increase the robustness of the results. All newborns were tested at term equivalent age and, to have a homogeneous population, we adopted strict inclusion criteria.

Despite having the same gestational age at the time of testing, their postnatal ages at test differ (for details see Table 1). Additionally, their exposure to voices, along with the unique auditory environment of the Neonatal Intensive Care Unit, could potentially impact their perception of the voice versus music contrast.

In our corpus, the contrast between full‐term and preterm newborns reported in Figures S8–S10, we analyzed the interaction effect of group and task. Only one PPI‐CAP showed a significant interaction effect (*p* = 0.0101). However, the subset of full‐term data was too small to draw conclusive results.

Finally, we did not measure the different sleep stages in our newborns as it would need contemporaneous EEG not feasible in newborn fMRI.

Further analyses with an increased sample size will allow us to evaluate the effect of prematurity on vocal and instrumental music perception. We only partially addressed the modulatory effect of voice or music familiarity, which will be the focus of future analyses. Data on prenatal exposure to music and singing voices will be essential for interpreting the overmentioned future analyses. Moreover, the clinical relevance of exposure to vocal versus instrumental music in preterm and newborn infants has yet to be fully explored and will be the focus of future research. Finally, due to the gray matter mask that restricted the analysis within the gray matter, we did not have the opportunity to explore the contribution of deep brain regions into the obtained dynamic PPI‐CAP patterns. Future prospective of this study will include the use of a whole‐brain mask allowing the exploration of deep brain regions (e.g., subcortical regions).

## AUTHOR CONTRIBUTIONS

Serafeim Loukas: formal analysis, methodology, visualization, methods conceptualization, writing‐original draft, writing‐review & editing. Manuela Filippa: conceptualization, data acquisition, writing‐original draft, writing‐review & editing. Joana Sa de Almeida: data acquisition, initial analysis, writing‐review & editing. Andrew S. Boehringer: visualization, review & editing. Cristina Borradori Tolsa: review & editing. Francisca Barcos‐Munoz: review & editing. Didier M. Grandjean: writing‐review & editing, supervision. Dimitri van de Ville: writing‐review & editing, supervision. Petra S. Hüppi: conceptualization, writing‐review & editing, supervision, project administration, resources, funding acquisition.

## CONFLICT OF INTEREST STATEMENT

All the authors declare that they have NO affiliations with or involvement in any organization or entity with any financial or non‐financial interest in the subject matter or materials discussed in this manuscript.

## Supporting information


**DATA S1.** Supporting information.

## Data Availability

All data were acquired in the context of the research project approved by the ethical committee. The participant consent form did not include any clause for reuse or sharing of data. It means that all data (clinic, biologic and imaging) cannot be used with any other aim apart from the present research study and would not be shared with third parties.
