# Supplementary material for: Newborn's neural representation of instrumental and vocal music as revealed by fMRI: A dynamic effective brain connectivity study
Source: Hum Brain Mapp. 2024 Jul 12;45(10):e26724. doi: 10.1002/hbm.26724 (PMC11245569; doi:10.1002/hbm.26724)
Supplement: Supplementary file 1 — DATA S1. Supporting information. [file HBM-45-e26724-s001.docx]

**Supplementary Material**

**
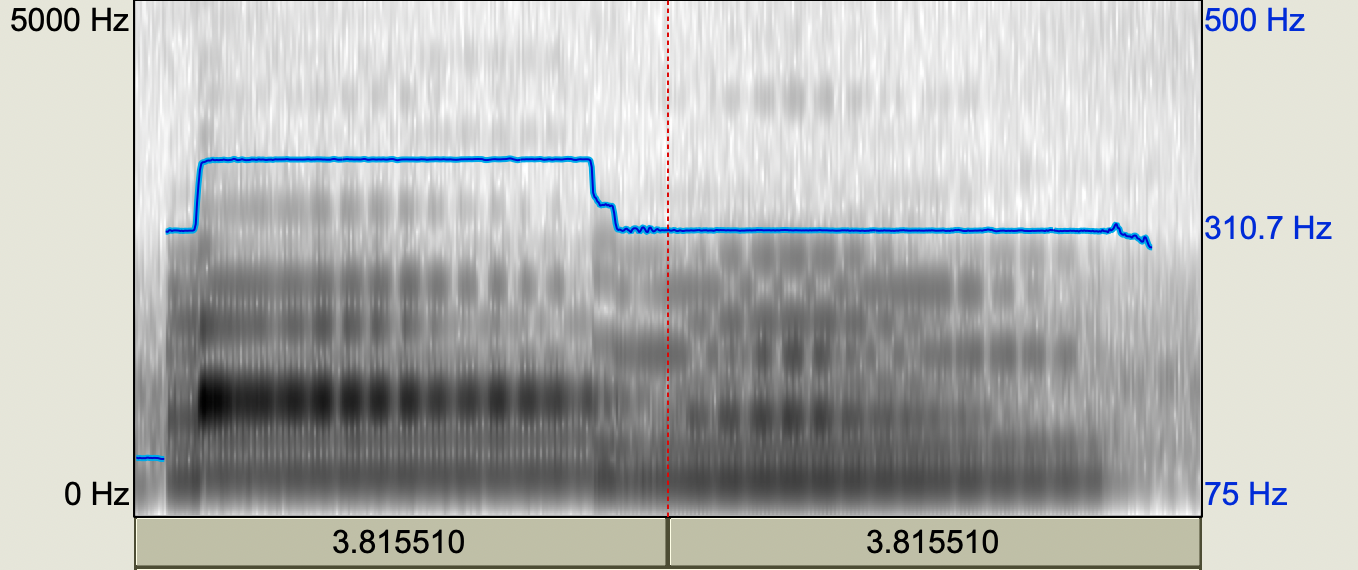
**

**Figure S1: Spectrogram with pitch, Extract 1**

**
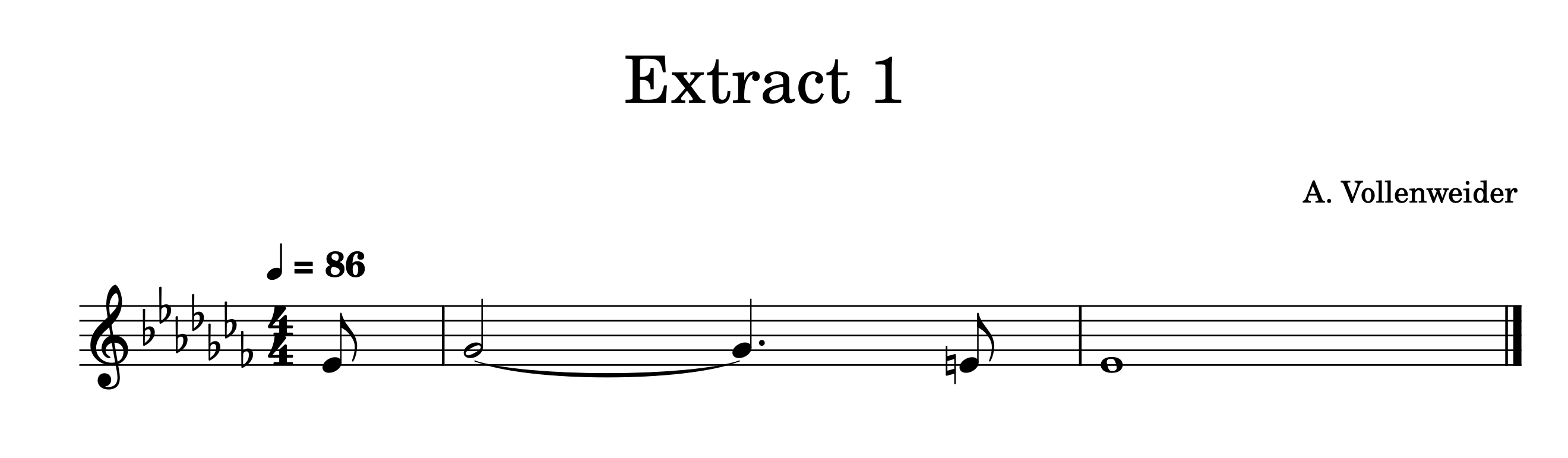
**

**Figure S2: Music Sheet, Extract 1**

**
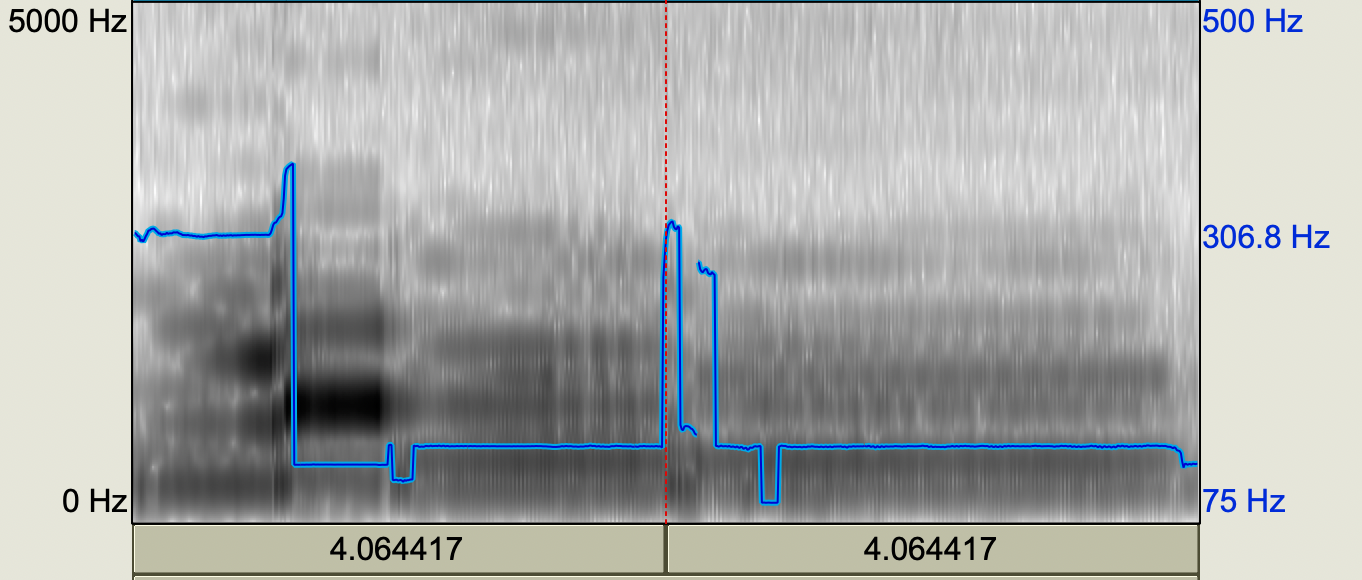
**

**Figure S3: Spectrogram with pitch, Extract 2**

**
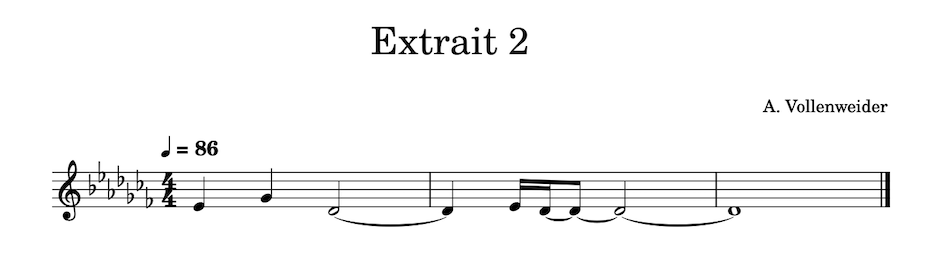
**

**Figure S4: Music Sheet, Extract 2**


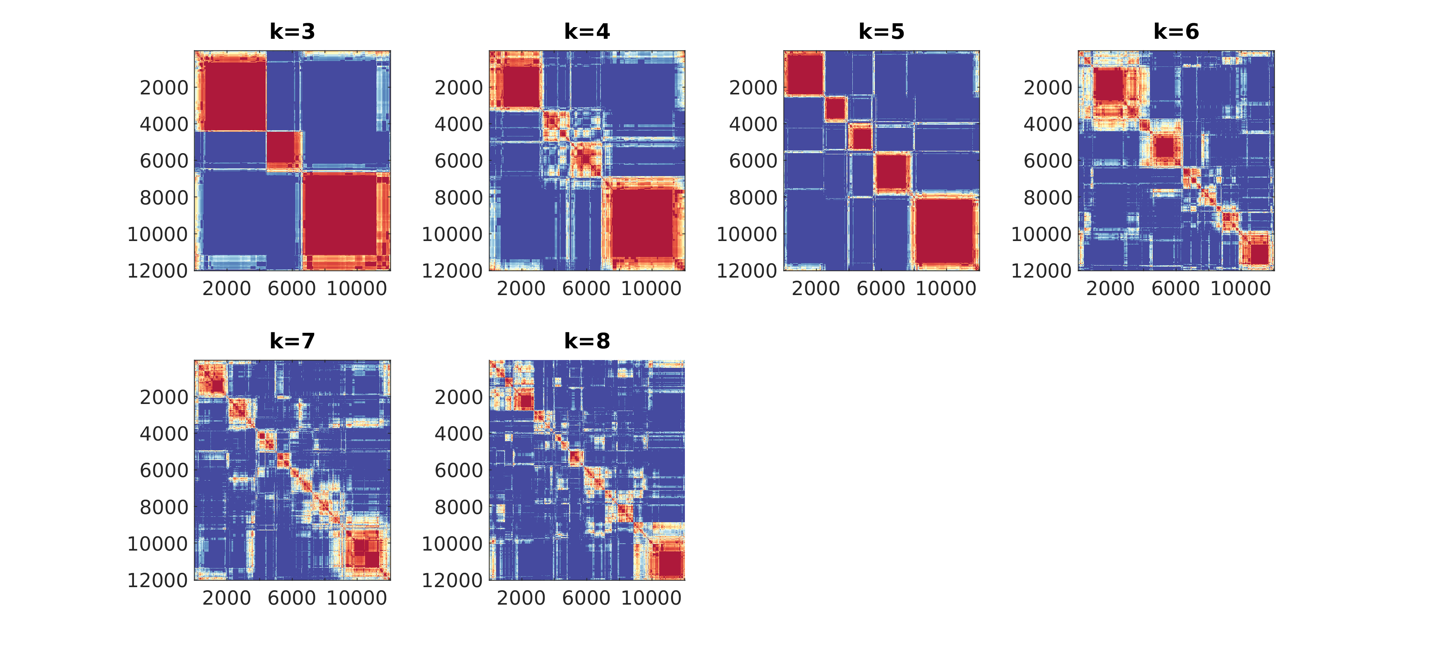
**Figure S5**: **Optimal number of clusters for the auditory-seed PPI-CAPs analysis.** The consensus matrices show that the most stable value is k = 5 (i.e., k for which the fMRI frames would most consistently be clustered together or separately). The colormap represents the proportion of time when two frames are consistently clustered either together (both in the same cluster; red) or separately (never in the same cluster; blue).


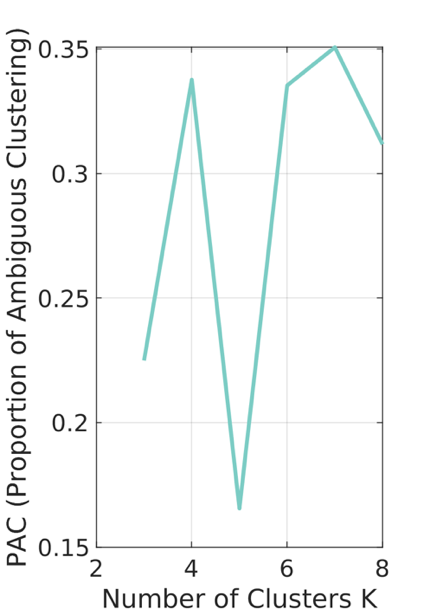


**Figure S6**: **The Proportion of Ambiguously clustered frames metric (PAC).** The PAC metric is a robust way to assess clustering performance. The PAC metric is the lowest for k=5 confirming that this is the optimal number of clusters for our data. In a perfect clustering scenario, the consensus matrix would consist of only 0s and 1s, and the PAC assessed on the (0, 1) interval would have a perfect score of 0.

**
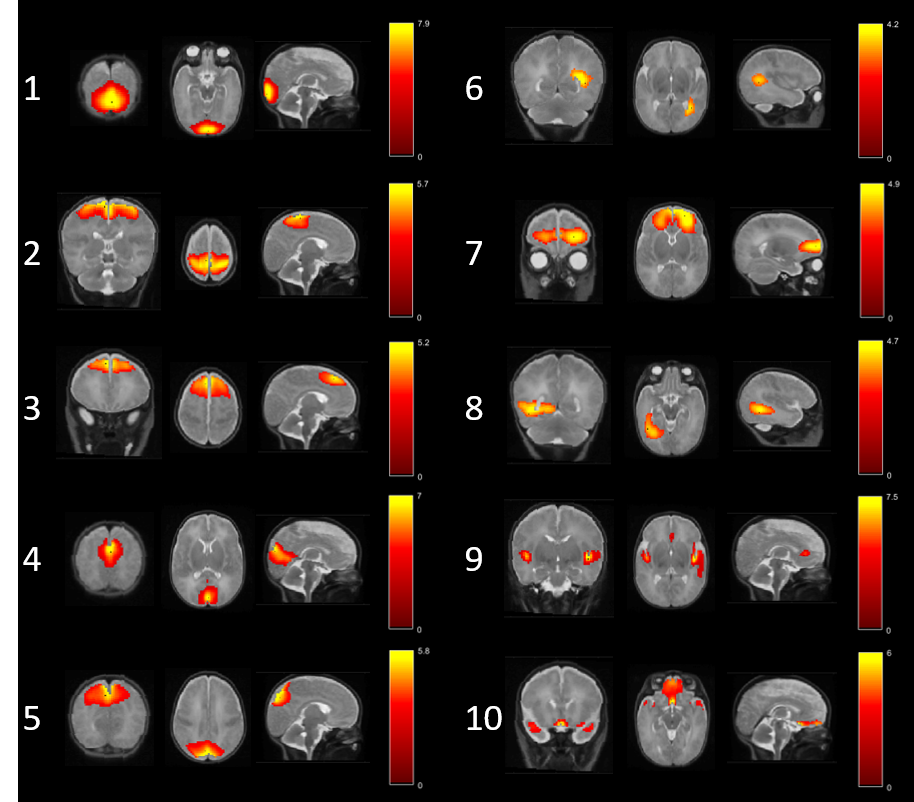
**

**Figure S7: The ICA networks as derived in Lordier et al., 2019.** Visual, Sensorimotor, Superior frontal, Posterior Cingulate Cortex, Precuneus, Right posterior temporal cortex (RpTG), superior frontal, left posterior temporal cortex (LpTG), Salience, Orbitofrontal (OFC). For a detailed description of these networks refer to Lordier et al., 2019**.**

**
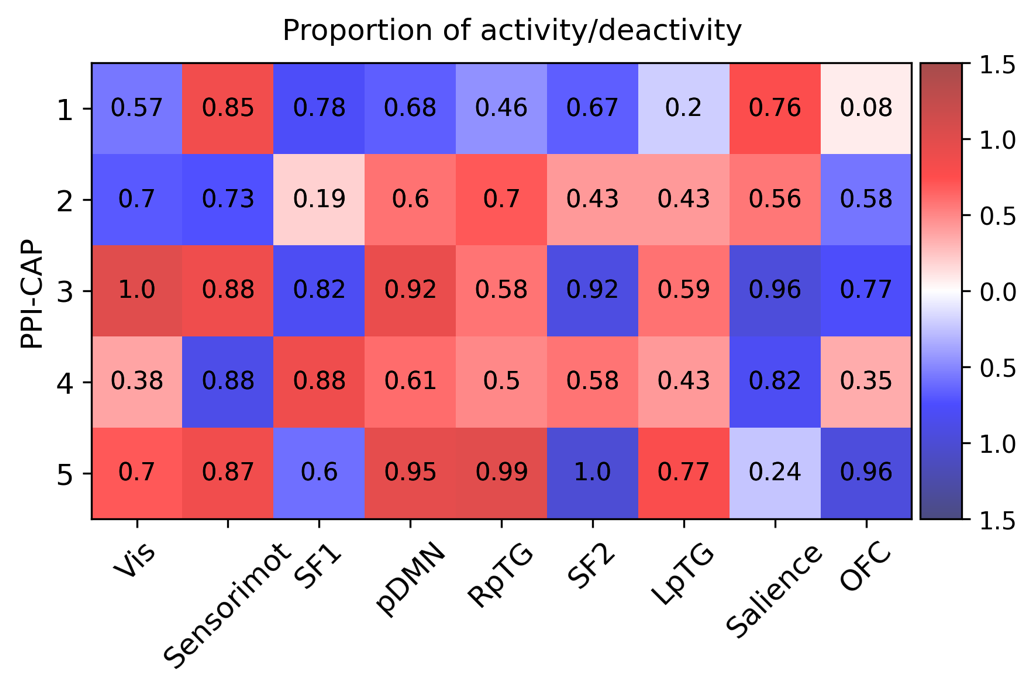
**

**
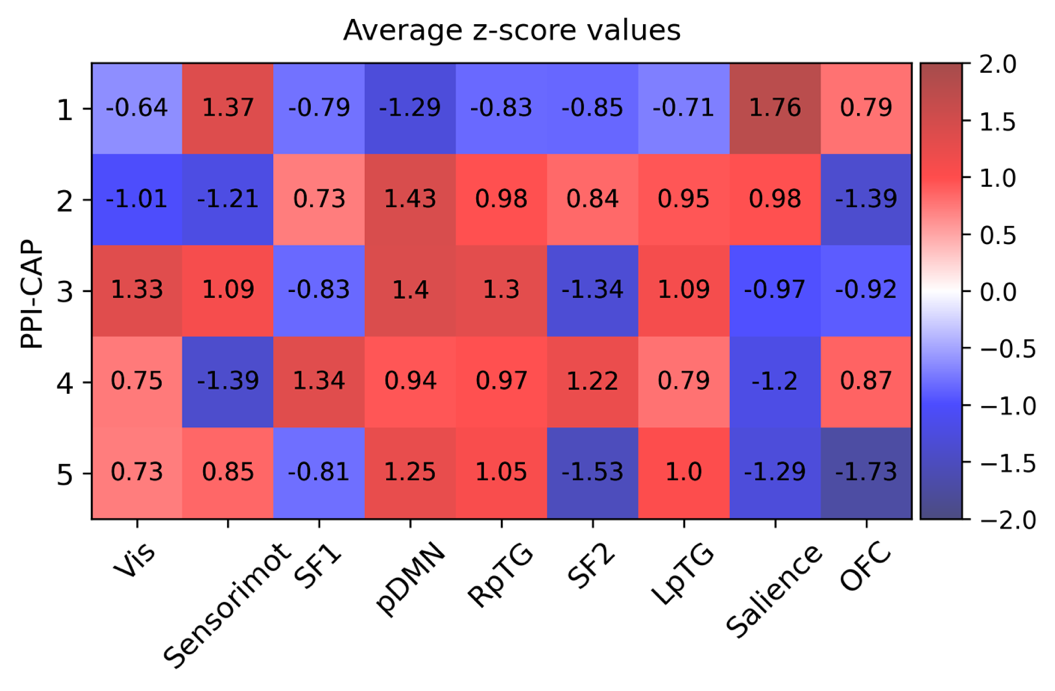
**

**Figure S8: Network assignment.** **Upper panel**: The proportion of activation or deactivation of the eleven ICA networks (each column) was estimated to define an activity “profile” for each PPI-CAP (each row). High proportion of activation is represented by red colors (1 means 100% of the network was active). High proportion of deactivation is represented by blue colors (1 means 100% of the network was deactivated). **Lower panel**: The average (within network) z-score values for each ICA component (column) overlayed onto each PPI-CAP map (row).


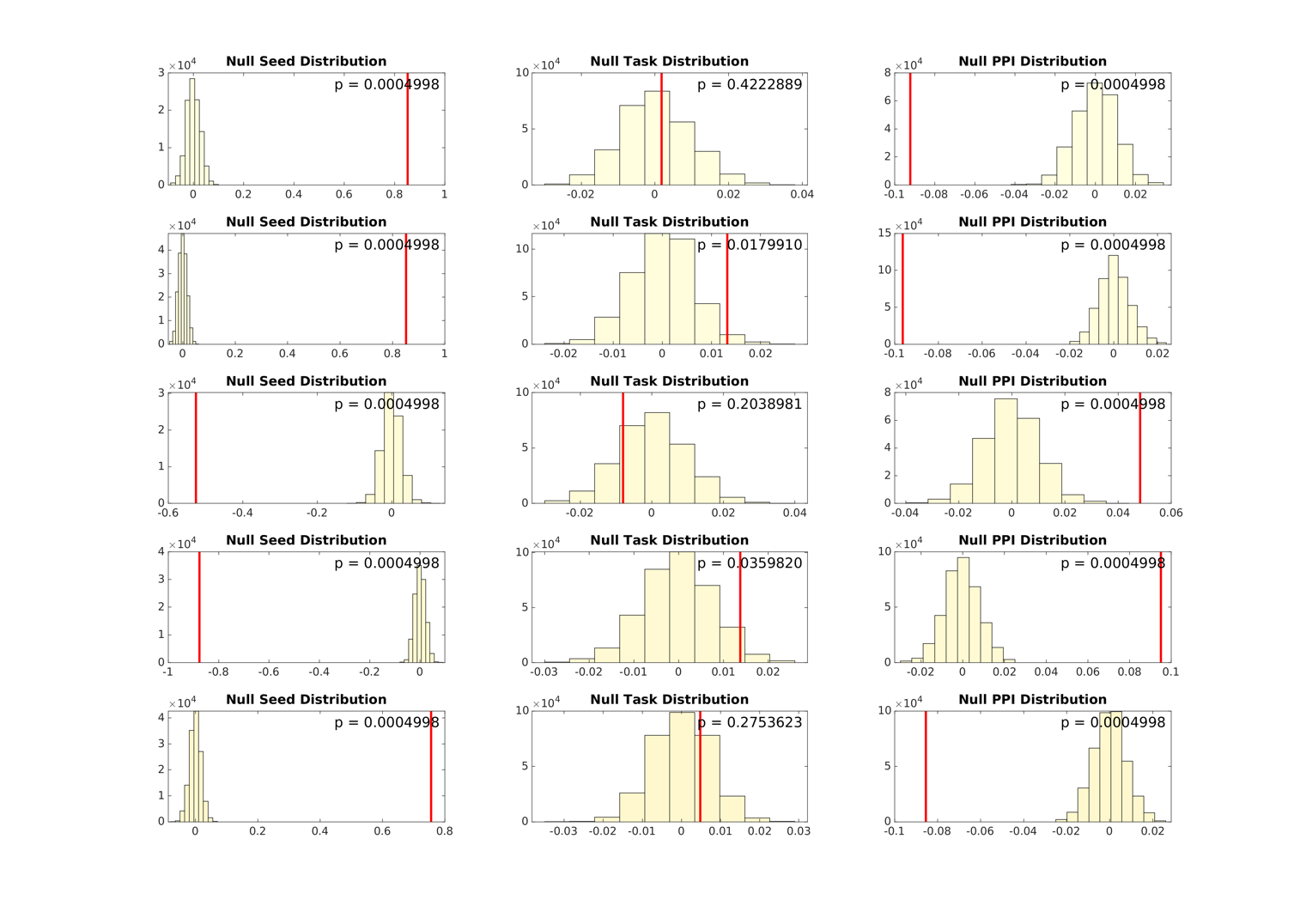


**Figure S9: Significance assessment of the PPI-CAP effects.** Each histogram illustrates the distribution of determinant values of the confusion matrices obtained from 2000 random permutations of the frame labels composing each PPI-CAP, with respect to seed, task or PPI effect, respectively. The red line indicates where the determinant of the confusion matrix for real data lies.

 
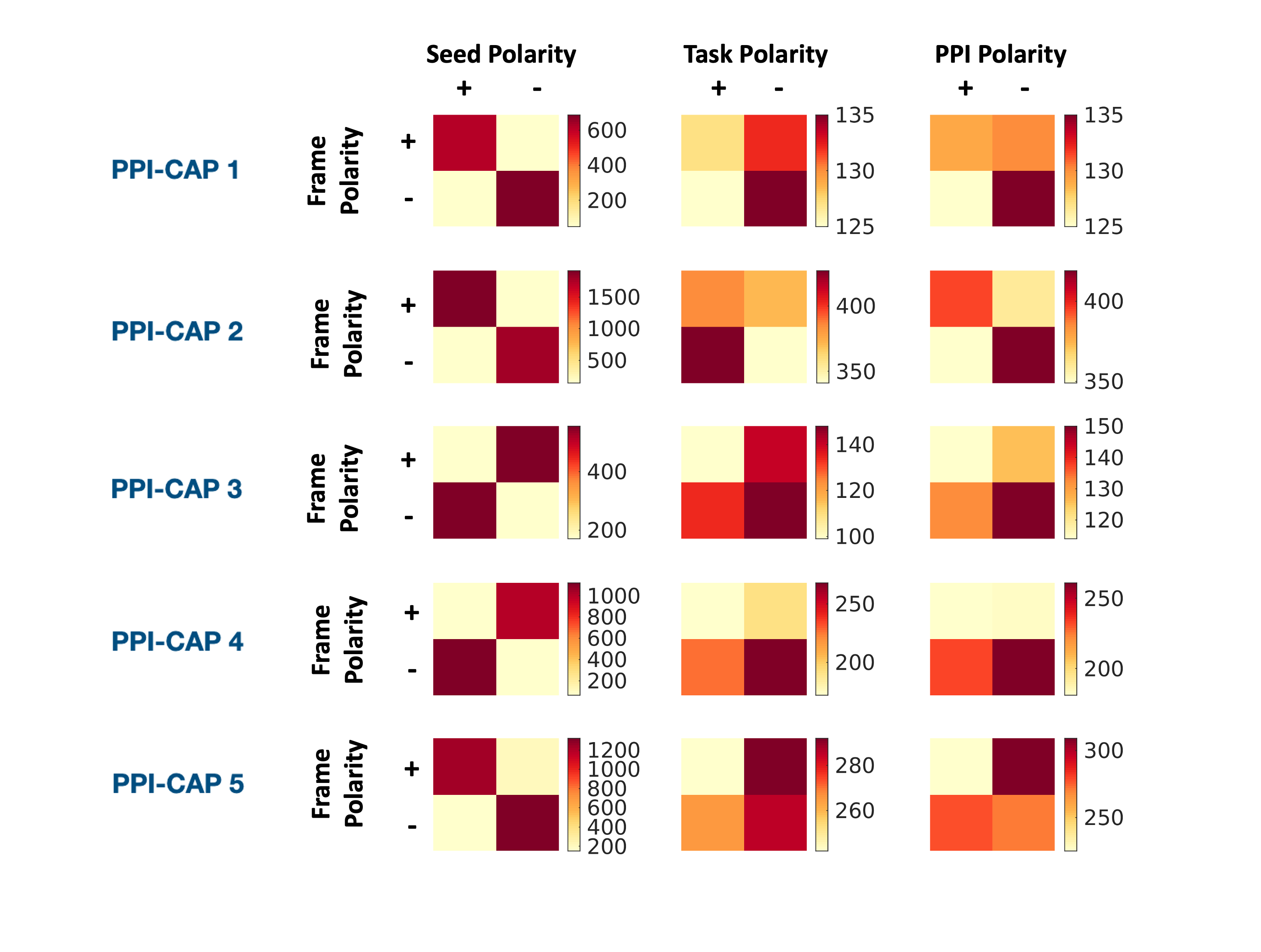


**Figure S10: Confusion matrices of main and interaction effects for mother’s vs stranger’s voice.** Each row corresponds to a PPI-CAP and each column to a specific effect (seed, task, or PPI effect, respectively). Main and interaction effects can be revealed using confusion matrices that depict how often the sign of a PPI-CAP switches in the same way as each of the underlying effects. The signs for each effect were defined as follows: Seed — positive and negative signs correspond to frames when the seed (auditory cortex) was activated or deactivated, respectively; Task — positive signs correspond to the mother’s singing condition while negative signs correspond to moments of the stranger’s singing; PPI — Interaction signs are calculated as element- by-element multiplication of the main effect signs. Light yellow indicates the lowest number of frames, while dark red indicates the highest number of frames. Null distribution and the exact p-values of these effects are shown in Figure S7.

 
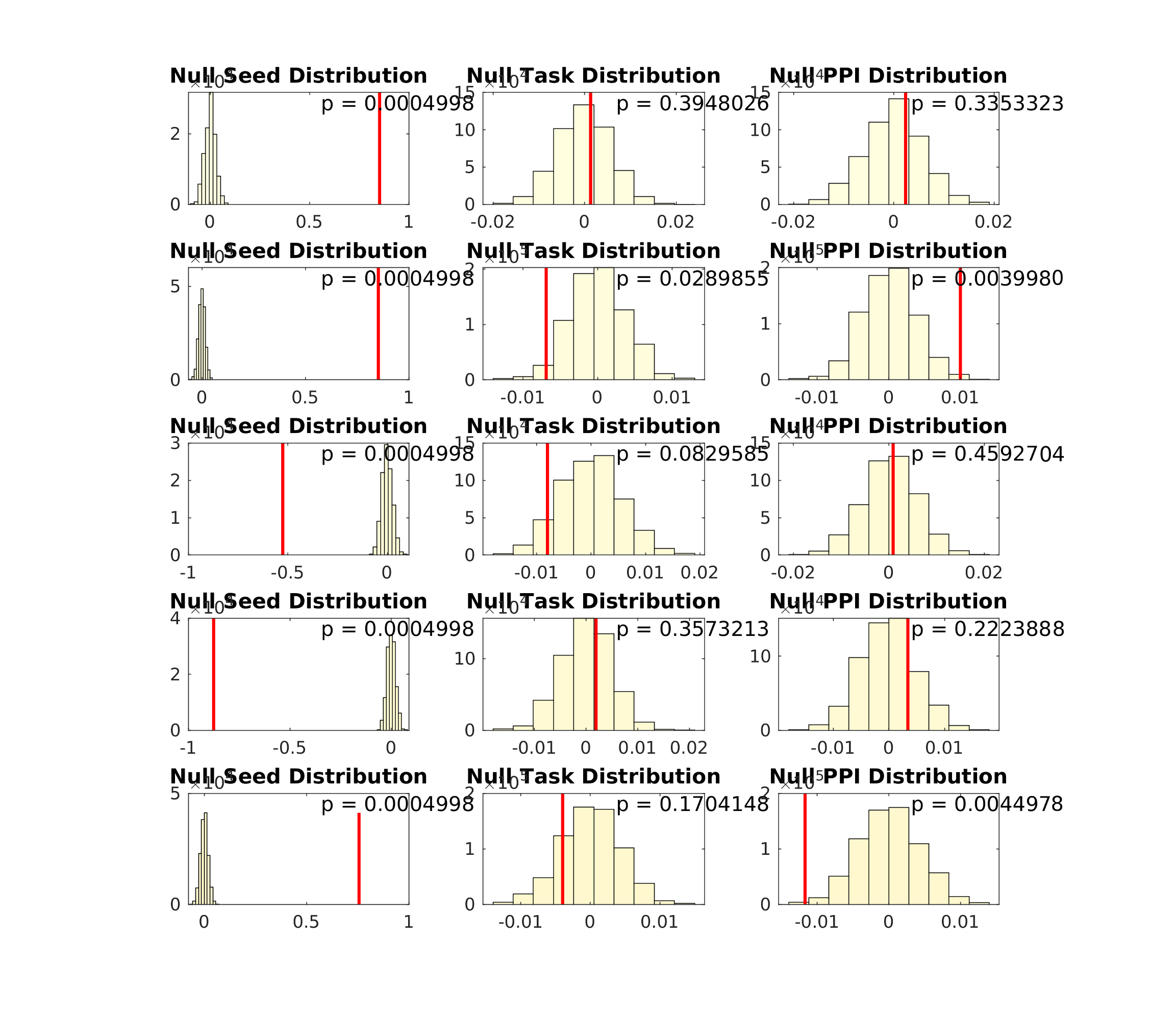
**Figure S11a: Significance assessment of the PPI-CAP effects of mother’s vs stranger’s voice.** Each histogram illustrates the distribution of determinant values of the confusion matrices obtained from 2000 random permutations of the frame labels composing each PPI-CAP, with respect to seed, task, or PPI effect, respectively. The red line indicates where the determinant of the confusion matrix for real data lies. Only PPI-CAP 2 and 5 has a significant group*task interaction effect (respectively p = 0.0034 and p= 0.0045), and for the analyses we used the same dataset as for the main manuscript.

Mother’s voice Stranger’s voice

**
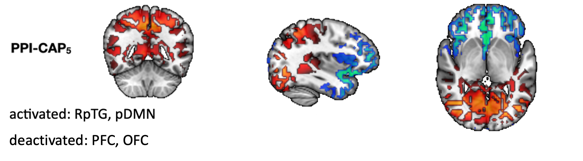

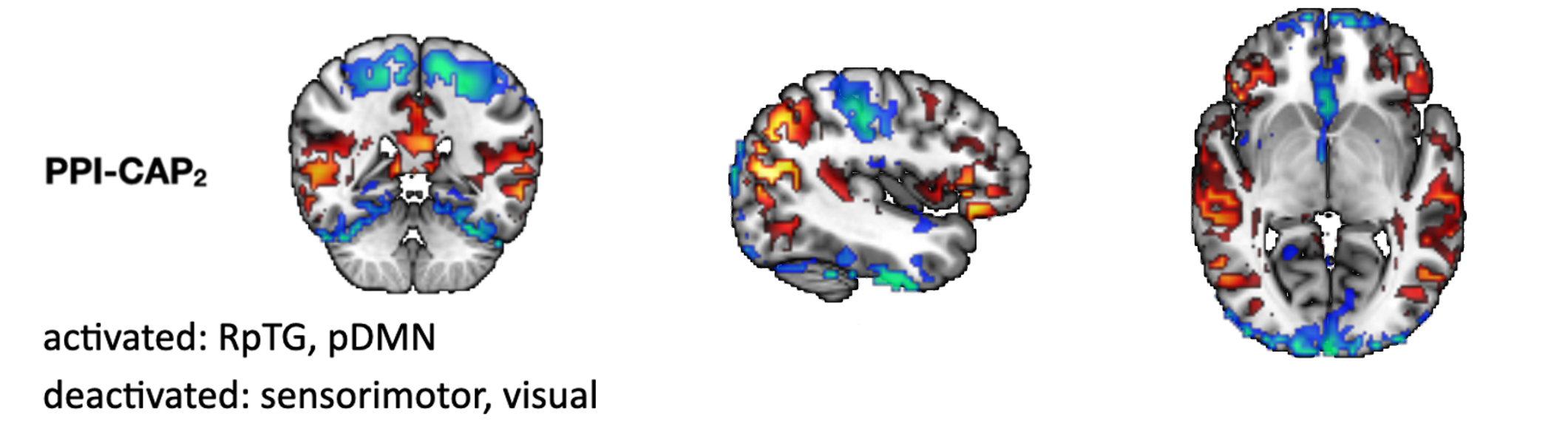
**

**Figure S11b: Result summary of the PPI-CAP effects of mother’s vs stranger’s voice**

 
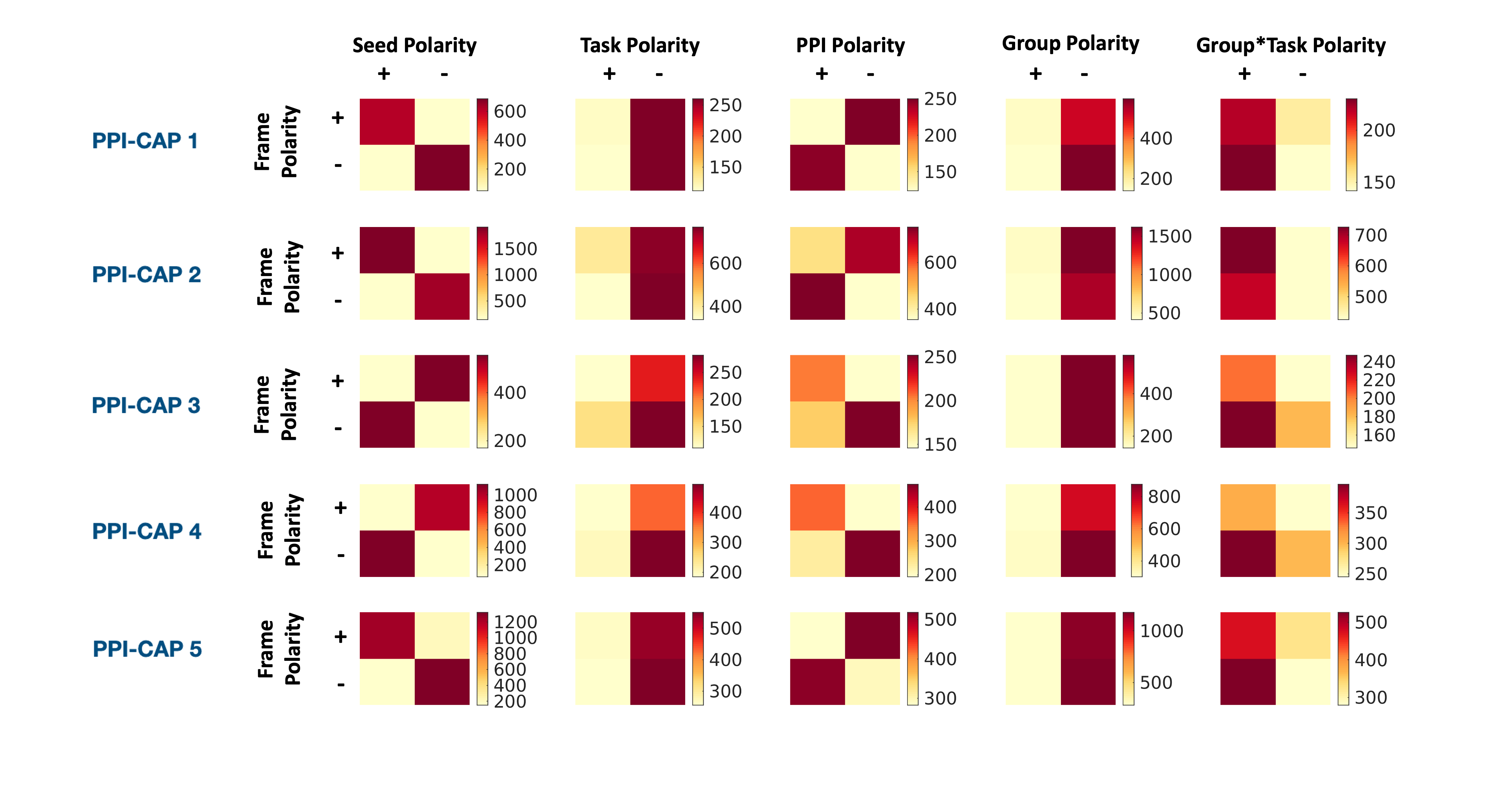


**Figure S12: Confusion matrices of main and interaction effects of music vs singing for full-term vs preterm groups.** Each row corresponds to a PPI-CAP and each column to a specific effect (seed, task, PPI, group, and group*task interaction effects, respectively). Main and interaction effects can be revealed using confusion matrices that depict how often the sign of a PPI-CAP switches in the same way as each of the underlying effects. The signs for each effect were defined as follows: Seed — positive and negative signs correspond to frames when the seed (auditory cortex) was activated or deactivated, respectively; Task — positive signs correspond to the Instrumental music condition while negative signs correspond to moments of Singing Condition; PPI — Interaction signs are calculated as element- by-element multiplication of the main effect signs; Group — Positive signs correspond to full term and negative signs to preterm; Group*Task – Interaction signs are  calculated as element- by-element multiplication of the group and task effect signs. Light yellow indicates the lowest number of frames, while dark red indicates the highest number of frames. Null distribution and the exact p-values of these effects are shown in Figure S9.


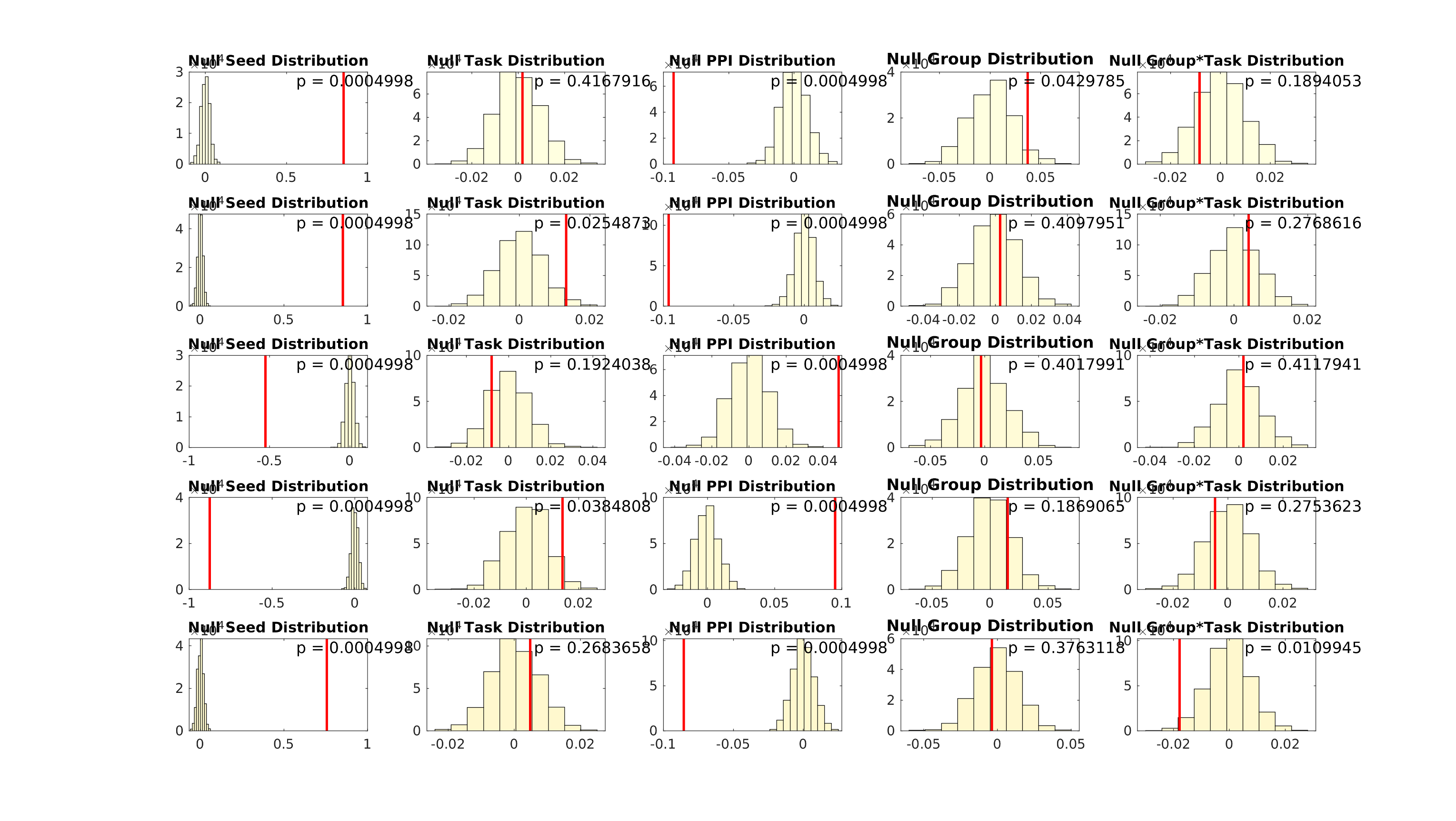


**Figure S13: Significance assessment of the PPI-CAP effects of music vs singing for full-term vs preterm groups.** Each histogram illustrates the distribution of determinant values of the confusion matrices obtained from 2000 random permutations of the frame labels composing each PPI-CAP, with respect to seed, task, PPI, and group effect, respectively. The final column indicates the interaction effect of group and task between full-term and preterm newborns. The red line indicates where the determinant of the confusion matrix for real data lies. Only PPI-CAP 5 has a significant group*task interaction effect (p = 0.0101), however the full-term data subset is too small to be conclusive (n = 10 and n = 35, for full-term and preterm, respectively).


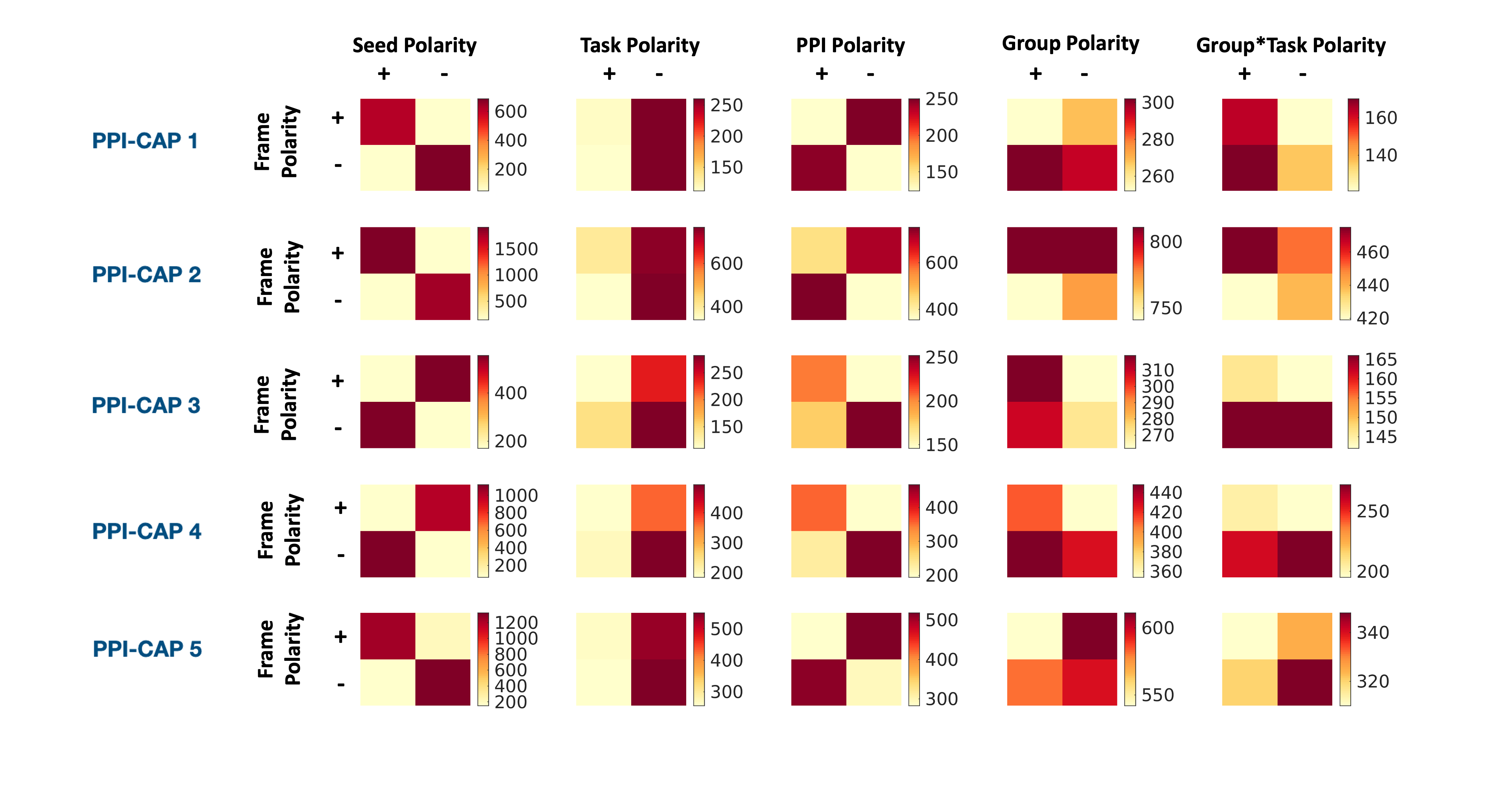


**Figure S14: Confusion matrices of main and interaction effects of music vs singing for preterm controls vs preterm music groups.** Each row corresponds to a PPI-CAP and each column to a specific effect (seed, task, PPI, group, and group*task interaction effects, respectively). Main and interaction effects can be revealed using confusion matrices that depict how often the sign of a PPI-CAP switches in the same way as each of the underlying effects. The signs for each effect were defined as follows: Seed — positive and negative signs correspond to frames when the seed (auditory cortex) was activated or deactivated, respectively; Task — positive signs correspond to the Instrumental music condition while negative signs correspond to moments of Singing Condition; PPI — Interaction signs are calculated as element- by-element multiplication of the main effect signs; Group — Positive signs correspond to preterm controls and negative signs to preterm music; Group*Task – Interaction signs are  calculated as element- by-element multiplication of the group and task effect signs. Light yellow indicates the lowest number of frames, while dark red indicates the highest number of frames. Null distribution and the exact p-values of these effects are shown in Figure S11.


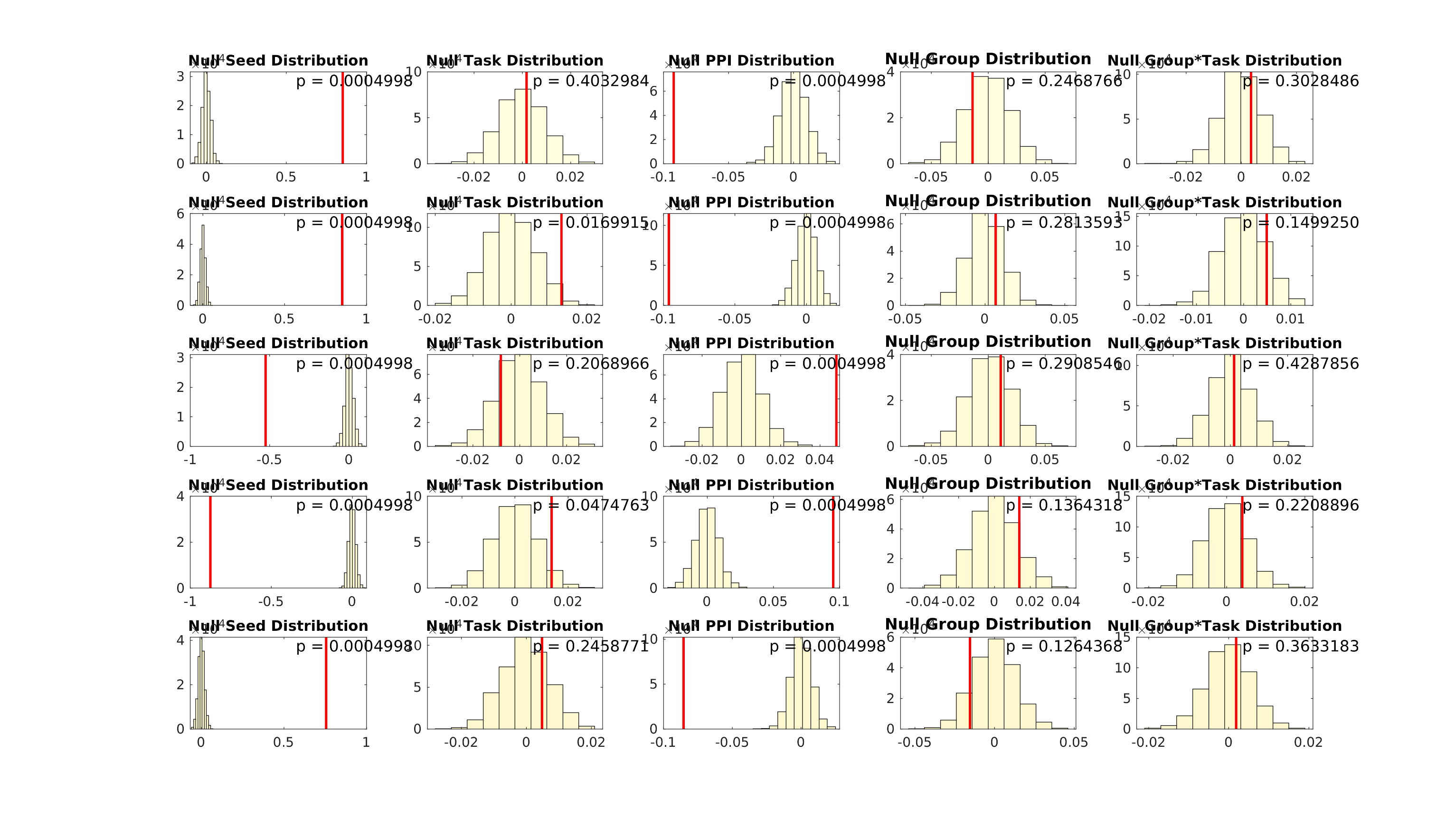


**Figure S15: Significance assessment of the PPI-CAP effects of music vs singing for preterm controls vs preterm music.** Each histogram illustrates the distribution of determinant values of the confusion matrices obtained from 2000 random permutations of the frame labels composing each PPI-CAP, with respect to seed, task, PPI, and group effect, respectively. The final column indicates the interaction effect of group and task between preterm controls and preterm music newborns. The red line indicates where the determinant of the confusion matrix for real data lies. None of the PPI-CAPs have a significant group*task interaction effect, however the data subsets are too small to be conclusive (n = 17 and n = 18, for preterm control and preterm music, respectively).

**Table S1:** Anatomical region information of the ICA components as defined in Lordier et al., 2019.

| Number of the component | RSN | Abbreviations | Anatomical location |
| --- | --- | --- | --- |
| 1 | Visual | Vis | Bilateral occipital lobe |
| 2 | Sensorimotor | SM | Bilateral precentral gyrus  Bilateral postcentral gyrus |
| 3 | Superior Frontal | SF | Bilateral medial superior frontal cortex |
| 4 | Posterior cingulate cortex | PCC | Bilateral precuneus gyrus  Posterior cingulate cortex |
| 5 | Precuneus | Prec | Bilateral Precuneus |
| 6 | Right posterior temporal cortex | RpTG | Right posterior superior temporal gyrus  Right posterior middle temporal gyrus |
| 7 | Prefrontal Cortex | PFC | Bilateral Prefrontal Cortex |
| 8 | Left posterior temporal | LpTG | Left posterior superior temporal gyrus  Left posterior middle temporal gyrus |
| 9 | Salience | Salience | Insula  Perigenual cingulate cortex |
|  | Thalamus | Thal | Mesencephalum  Thalamus : anteroventral thalamus, ventral anterior thalamic nucleus, anterior thalamus nucleus  Dorsal anterior cingulate cortex |
| 10 | Orbitofrontal | OFC | Medial orbital cortex and gyrus rectus  Inferior temporopolar region  Planum polare |
